# Prevalence of Stress, Anxiety, and Depression Among Pregnant Women in Jeddah

**DOI:** 10.7759/cureus.27174

**Published:** 2022-07-23

**Authors:** Maryam A Khouj, Samera Albasri, Anas A Albishri, Shadi M Softa, Alanoud S Almaslamani, Hanin M Ahmad

**Affiliations:** 1 Medicine, King Abdulaziz University, Jeddah, SAU; 2 Faculty of Medicine, King Abdulaziz University, Jeddah, SAU; 3 Obstetrics and Gynecology, King Abdulaziz University Hospital, Jeddah, SAU; 4 College of Medicine, King Abdulaziz University, Jeddah, SAU

**Keywords:** mental illness in pregnancy, pregnancy during pandemic, psychological impact of pregnancy, perinatal anxiety screening scale, depression anxiety stress scale

## Abstract

Objectives: To estimate the prevalence of depression, anxiety, and stress among pregnant women living in Jeddah, Saudi Arabia, and to assess associated risk factors.

Methods: The study, which was a cross-sectional design, was conducted under the supervision of King Abdulaziz University Hospital, a tertiary care center in Jeddah, Saudi Arabia, in May and June of 2021. The sample consisted of 200 pregnant women who completed an online questionnaire that included sociodemographic variables, obstetric information, pregnancy-related depression, stress, and anxiety symptoms, which were evaluated using the Depression Anxiety Stress Scale (DASS-21) and the Perinatal Anxiety Screening Scale (PASS).

Results: DASS-21 scores indicated the prevalence of depression, anxiety, and stress were 37.5%, 54.0%, and 25.0%, respectively. The PASS revealed that 29.5% of participants had minimal anxiety symptoms, 44.5% had mild-to-moderate anxiety symptoms, and 26.0% had severe anxiety symptoms. The three psychological health conditions were significantly associated with family/husband support, history of caesarean section, parity, and abortion.

Conclusions: Pregnant women should be screened routinely for any psychological disturbances, and women who are at high risk for mental illness should receive proper psychological care. Pregnant women, their families, and members of society should receive health-related education in order to prevent prenatal psychological issues as much as possible.

## Introduction

Pregnancy is considered a sanctified spiritual event for women in many countries, specifically in developing countries where they get enormous respect for being pregnant [1]. However, pregnancy is a complex and highly emotional period in the lives of most women. There are many transformations that occur during this period other than the observed physiological ones, including psychological and social effects. Mothers could start experiencing these changes from the very beginning of pregnancy until the postpartum period. Every new mother is prone to encountering frequent mood changes and emotional disturbances such as stress and/or mixed anxiety-depressive symptoms. Compromised maternal mental health in the perinatal period may result in physical complications for the newborn [2]. Maternal stress is associated with adverse effects on pregnancy, including preeclampsia, preterm birth, low birth weight, and neonatal morbidity [3]. Prenatal depression and anxiety are also associated with preterm labor and, hence, with low birth weight; the latter also increases the risk of hypertension and pre-eclampsia, as well as the risk of cesarean section (C-section) delivery [4-5]. Multiple studies have also shown that prenatal anxiety and depression are related to an increased incidence of nausea and vomiting during pregnancy, increased childbirth-related fear, a greater number of visits to obstetrics and gynecology clinics, and decreased sleep quality [2,6].

In the past few years, there has been an increase in evidence that large numbers of pregnant women suffer from psychiatric disorders. A Swedish population-wide study reported that the percentage of expectant mothers who suffer from at least one psychiatric illness is approximately 14% [7]. A Turkish study that evaluated the state of pregnant women during different trimesters of pregnancy has observed that depression, anxiety, and defective sleep quality were higher in the third trimester compared to the first and second trimesters [8]. A 2020 Pakistani study that explored antenatal stress and its associations amongst pregnant Punjabi women also reported high-stress levels amongst 42.2% of their respondents and concluded that it was mostly associated with low-income status, non-involvement of the respondent in household decision-making, desire for a son rather than a daughter, and experience of birth complications in past pregnancies [9].

In Saudi Arabia, very few studies have been conducted on the psychological implications of pregnancy among expectant mothers. A study performed in the Eastern Province of the country estimated that the prevalence of depression was 26.8% and that of anxiety was 23.6% [10]. Given the lack of research, very little is known about the epidemiology of psychiatric diseases among pregnant women in Saudi Arabia and their associated risk factors. Therefore, this study aims to evaluate the prevalence of stress, anxiety, and depressive symptoms in pregnant women living in Jeddah, Saudi Arabia, and to assess associated risk factors.

## Materials and methods

Study design and setting

This cross-sectional study was conducted in May and June 2021 under the supervision of the Department of Obstetrics and Gynecology at King Abdulaziz University Hospital (KAUH), a tertiary center in Jeddah, Saudi Arabia. The respondents were all pregnant women who lived in Jeddah.

Sample 

Convenience sampling was used and a total of 200 pregnant women living in Jeddah responded. Women were first asked if they had previously been officially diagnosed with mental illness, and those who answered 'yes' were excluded.

Data collection

Data were collected through an anonymous online questionnaire created with Google Forms and distributed through social media outlets. The survey consisted of three parts. The first part inquired about participants’ sociodemographic characteristics and obstetric history, age, level of education, employment status, monthly income, and prior and current pregnancies and births (gravidity, gestational age, mode of delivery, and history of abortions). The second part consisted of the validated Arabic version of the Perinatal Anxiety Screening Scale (PASS), which is a 31-item self-report questionnaire scored on a four-point scale: not at all = 0, sometimes = 1, often = 2, and almost always = 3, with the total score ranging from 0 to 93 [11]. The PASS consists of four subscales: (1) excessive worry and specific fears; (2) perfectionism, control, and trauma; (3) social anxiety; and (4) acute anxiety and adjustment. The subscales describe symptoms of anxiety as they are manifested in perinatal women. A total PASS score is obtained by adding the scores of all the items, and a cut-off score of 26 is recommended for differentiating between high and low risk of perinatal anxiety.

The third part of the questionnaire consisted of the Depression Anxiety Stress Scale-21 (DASS-21), which has 21 items divided into three subscales with seven items each. The validated Arabic version of the DASS-21 was used to measure depressive symptoms (for example, feeling downhearted and blue), anxiety symptoms (for example, feeling close to panic), and general stress symptoms (for example, having a tendency to overreact to situations) [12]. The response options are scored on a four-point scale (0 = did not apply to me at all to 3 = applied to me all of the time). Higher scores indicate a higher level of psychological distress. Scores for depression, anxiety, and stress were calculated by adding the scores of the items on the respective subscales. The recommended cut-off scores for conventional severity labels (normal, moderate, severe) are as follows: depression (normal = 0-9, moderate = 14-20, and severe = 21-27); anxiety (normal = 0-7, moderate = 10-14, and severe = 15-19); and stress (normal = 0-14, moderate = 19-25, and severe = 26-33). Scores on the DASS-21 are multiplied by two to calculate the final score.

Research ethics 

This study was approved by the Research Ethics Committee of KAUH (reference number 13-21). Informed consent was obtained from all the respondents after the aim and objectives of the study were explained.

Statistical analysis

Microsoft Excel 2016 (Microsoft® Corp., Redmond, WA, USA) was used for data entry and the analyses were performed using IBM SPSS Statistics for Windows, version 26 (IBM Corp., Armonk, NY, USA). Means and standard deviations were calculated to describe continuous variables, and frequencies and percentages were calculated to describe categorical variables. A Student's t-test was used to evaluate differences between two continuous variables, and chi-square tests were used to evaluate associations among categorical variables. A p-value of <0.05 was considered statistically significant.

## Results

A total of 200 women (mean age = 32 ± 6.8 years) who were eligible for the study participated. Their sociodemographic information is presented in Table 1.

**Table 1:** Sociodemographic data of the participants (N=200)

		Frequency	Percentage
Age group (years)	≤20	5	2.5%
	21–30	79	39.5%
	31–40	96	48.0%
	≥40	20	10.0%
Education level	Primary school	0	0.0%
	Secondary school	1	0.5%
	High school	41	20.5%
	University	148	74.0%
	Masters	7	3.5%
	PhD	3	1.5%
Occupation	Yes	60	30.0%
	No	140	70.0%
Monthly income (Saudi Riyals)	<5000	28	14.0%
	5000–10,000	76	38.0%
	>10,000	96	48.0%
Spouse and family support	Yes	185	92.5%
	No	15	7.5%

The majority of the participants received higher education but were unemployed. Table 2 presents the obstetric histories of the participants.

**Table 2:** Obstetric data of the participants (N=200) C-section: caesarean section

		Frequency	Percentage
Gravidity	1–3	103	51.5%
	4–6	73	36.5%
	>6	24	12.0%
Parity	0–3	154	77.0%
	4–6	43	21.5%
	>6	3	1.5%
Abortion/miscarriage	0–2	185	92.5%
	3–4	11	5.5%
	>4	4	2.0%
Stage of current pregnancy	First trimester	48	24.0%
	Second trimester	73	36.5%
	Third trimester	79	39.5%
Preferred mode of delivery	Vaginal delivery	152	76.0%
	C-section	48	24.0%
Mode of last delivery	I have never given birth	37	18.5%
	Vaginal delivery	95	47.5%
	C-section	44	22.0%
	Assisted vaginal delivery	24	12.0%
History of C-section	Yes	57	28.5%
	No	143	71.5%
Was the C-section performed on your request?	Not applicable	143	71.5%
	Yes	6	3.0%
	No	51	25.5%

The most commonly received responses were from women experiencing their second pregnancies (20%), most of whom had never had a miscarriage or abortion. The majority of the women were in their third trimester, with the most common mode of last delivery being vaginal.

The PASS scores indicated that 41.5% of the women were at low risk of developing an anxiety disorder and 58.5% were at high risk (scores = 26-93). Most of the participants (44.5%) had mild-to-moderate anxiety symptoms, 29.5% were asymptomatic, and only 26.0% had severe symptoms. The scores of the four PASS subscales are listed in Table 3.

**Table 3:** Subscales of the PASS PASS: Perinatal Anxiety Screening Scale

	Mean	Standard deviation
Excessive worry and fear	11.60	6.31
Perfectionism	9.74	4.97
Social anxiety	4.55	3.57
Acute anxiety	5.72	4.98

The subscale with the highest mean score (11.6) was "Excessive Worry and Specific Fears," while the "Social Anxiety" subscale had the lowest mean score (4.5). The highest overall mean PASS score was found for women in their first trimester (35.4 ± 17.6), and the lowest was for women in their third trimester (29.1 ± 15.8). No significant association was found between PASS severity and age group (p = 0.659), occupation status (p = 0.802), educational level (p = 0.264), or monthly income (p = 0.983). However, a significant association was identified between the PASS severity score and support from the participants’ spouse and family (p = 0.016), mode of last delivery (p = 0.015), and history of C-section delivery (p < 0.001). An independent-samples t-test also revealed a significant relationship between the overall PASS score and the women’s preferred mode of delivery (p = 0.029).

The DASS-21 results indicate that 37.5% of participants had some level of depressive symptoms, with the lowest depression score in our sample being 0 in 33 of the women, while the highest was 38 in one woman. The severity of each of the depression, anxiety, and stress scores is illustrated in Table 4.

**Table 4:** Psychological outcomes determined by the DASS-21 (N = 200) DASS-21: Depression Anxiety Stress Scale-21

Psychological category		Frequency	Percentage
Depression severity	Normal	125	62.5%
	Mild depression	28	14.0%
	Moderate depression	25	12.5%
	Severe depression	11	5.5%
	Extremely severe depression	11	5.5%
Anxiety severity	Normal	92	46.0%
	Mild anxiety	20	10.0%
	Moderate anxiety	48	24.0%
	Severe anxiety	18	9.0%
	Extremely severe anxiety	22	11.0%
Stress severity	Normal	150	75.0%
	Mild stress	13	6.5%
	Moderate stress	22	11.0%
	Severe stress	9	4.5%
	Extremely severe stress	6	3.0%

The most frequently reported symptom of depression (64.0%) was "feeling down-hearted and blue," and the least reported symptom (30.5%) was "I felt I wasn’t worth much as a person." Anxiety symptoms were reported by 54.0% of the participants, of which the most repeated score was 4. The most reported anxiety-related symptom was experiencing breathing difficulties, which was reported by 66.5% of participants, whereas only 30.5% reported that they felt close to panicking during the past week. The least reported psychological state was stress, which was reported by only a quarter of the respondents, with the majority (17.5%) complaining of only mild-moderate symptoms. The most reported stress item (74.5%) was "I found it hard to wind down," and the least reported item (48.5%) was "I was intolerant of anything that kept me from getting on with what I was doing."

No significant association was found between age and any of the three psychological variables (depression, p = 0.854; anxiety, p = 0.467; and stress, p = 0.463). A higher prevalence of depression, anxiety, and stress was reported among university graduates than among women with any other educational level. The chi-square test did not reveal any association between the severity of anxiety (p = 0.419) or stress (p = 0.967) and the three psychological problems. However, the depression severity was associated with the monthly income (p = 0.049), in which people of higher incomes had overall more severe depression levels. Also, there was no association with employment status (depression, p = 0.130; anxiety, p = 0.992; stress, p = 0.679). A statistically significant relationship was detected between parity and depression (p = 0.025) and stress scores (p = 0.01). The mean scores on both the stress and depression scales were higher among multiparous women, followed by primiparous women, while nulliparous women had the lowest scores. Another significant relationship was found between the history of abortion/miscarriage and depression scores (p = 0.024); the greater the number of abortions or miscarriages, the lower the depression score. Furthermore, significant associations were found between support from the participants’ husbands/families and the severity of depressive (p = 0.001), anxiety (p = 0.004), and stress (p < 0.001) symptoms. A one-way analysis of variance (ANOVA) also revealed significant differences among means of the mode of the last delivery and depression (p = 0.02), anxiety (p = 0.021), and stress (p < 0.001) scores. A higher overall mean DASS-21 score was observed among women who underwent a C-section as their last mode of delivery (40.6 ± 27.25), followed by those who underwent assisted vaginal delivery (30.25 ± 24.37), those who underwent vaginal delivery (28.12 ± 22.40), and those who had never given birth before (20.59 ± 14.61). A history of delivery via C-section was also significantly associated with the severity of depression (p = 0.007), anxiety (p = 0.03), and stress (p = 006) symptoms. No significant relationship was found between stress and anxiety severity or whether a C-section was performed without a medical indication. However, women who underwent C-sections at their own request had a lower level of depression than those who had a medical indication (p = 0.007). Furthermore, there was no significant relationship between participants’ preferred mode of delivery and the severity of any of the psychological problems (depression, p = 0.506; anxiety, p = 0.097; stress, p = 0.153).

Finally, the severity of the overall score of the PASS was significantly associated with the overall DASS-21 score (p < 0.001), as well as with the severity of perinatal anxiety and the severity of anxiety on the DASS-21 subscale (p < 0.001).

## Discussion

This study evaluated the prevalence of stress, anxiety, and depression and explored the associated risk factors among pregnant women visiting prenatal care clinics in Jeddah. In this study, 58.5% of the pregnant women who were surveyed were at a high risk of developing an anxiety disorder (according to the PASS), which is slightly higher than the proportion (43.5%) reported by Akinsulore et al. [13]. Furthermore, 70.5% of the participants reported anxiety symptoms; most (44.5%) had only mild-moderate symptoms, and 26.0% had severe symptoms. Sapkota et al. obtained similar results, reporting that 42.1% of pregnant women had mild-moderate anxiety symptoms and only 16.0% had severe symptoms [14]. The DASS-21 indicated that 37.5% of the participants had some level of depressive symptoms, more than half (54.0%) had anxiety symptoms, and only a quarter had any level of stress. Mild-to-moderate symptoms of depression, anxiety, and stress were reported by 26.5%, 34.0%, and 17.5% of participants, respectively. A study conducted during the coronavirus (COVID-19) pandemic reported a similar rate of depression (44.6%), and several other studies reported an increased prevalence of depression, ranging from 34.2% to 37.0% [15-16]. In contrast, many studies from before COVID-19 reported a lower prevalence of depression in pregnant women, including a Spanish study ranging from 21.4% to 23.4% depending on the trimester and a New Zealand study that reported a rate of 22% [17-18]. Thus, the current pandemic appears to have had a significant psychological impact on pregnant women worldwide.

Furthermore, the prevalence of anxiety in this study is similar to the results of the Indian study by Sanchana et al., which reported a prevalence of 40.5% and a Canadian study that reported a prevalence of 57% [15,19]. However, the prevalence of stress in this study was slightly lower than the 37.8% reported by Sanchana et al. [15]. Moreover, an Iranian study on stress levels reported a prevalence of 32.7% [5]. The difference in results may be attributable to the use of different stress scales in each study, which might yield different rates.

No significant relationships were found between several of the sociodemographic variables (for example, age, educational level, income, and occupation) and each of the three psychological problems investigated, which is similar to the results of Sanchana et al. [15]. Similarly, Fu et al. in China did not detect relationships between age and these three psychological variables [20]. Contrarily, Shrestha and Pun found a relationship between age and anxiety and reported that the younger a woman was, the higher the chances were of developing anxiety symptoms [21]. Conversely, an Iranian study found that older women had a higher level of anxiety [22]. This difference may be due to the varying age ranges of the samples in different studies. Furthermore, lack of experience and not knowing what to expect early on in motherhood might have resulted in the high prevalence of prenatal anxiety among younger women, while the higher prevalence of anxiety among older women could be attributed to multiple bad obstetric experiences in the past.

Unlike this study, a study at Imam Abdulrahman Bin Faisal University in Dammam, Saudi Arabia, found a relationship between educational level and depression, which could also be attributed to the use of different depression scales [10]. Furthermore, a Nigerian study reported that pregnancy-related anxiety was significantly associated with education and that women with low levels of education were more likely to suffer from anxiety [13].

Occupation status also appears to influence stress and anxiety. A study in Chongqing, China, revealed that housewives or women not working during pregnancy had a higher risk of perinatal stress and anxiety compared with those who kept working [23]. This could be because unemployed women have more time to think about their pregnancy, whereas employed women have more on their minds. In this study, 92.5% of the participants had the support of their husbands and families. In contrast, Bawahab et al. reported that almost half (45%) of the participants in their study experienced a lack of support at home [24]. Naturally, support from spouses/families may help women to endure their pregnancies with minimal psychological problems.

Regarding obstetrics, a significant association was not found between trimesters and psychological outcomes. However, a study in Zhoushan, China, found that the prevalence of both anxiety and depression was highest in the first trimester and lowest in the second trimester, according to the Self-Rating Anxiety Scale and Self-Rating Depression Scale [25]. Another Turkish study found that a larger number of women had depressive symptoms in their third trimester [8]. These disparities may be attributable to the assessment tools used and differences in study settings and populations. The association found between gravidity and anxiety (p < 0.05) was similar to that in an Indian study and contrary to that in the study by Sapkota et al. [14-15]. Moreover, parity was related to depression (p = 0.025) and stress (p = 0.01). The mean scores on both scales were highest among multiparous women, which corresponds to the results of Bawahab et al., who stated that having more than three daughters increased the likelihood of depression almost four-fold [24]. Furthermore, a significant relationship was found between the history of abortion or miscarriage and depression (p = 0.024). The larger the number of abortions or miscarriages, the lower the depression score. This could be because women who have suffered from more miscarriages do not get as severe of a psychological strain as those who are suffering from it for the first time. However, anxiety (DASS-21) was not significantly related to abortion history (p = 0.091). In contrast, a study by Bawahab et al., which included 119 women who had previous abortions, found that most of them (92.4%) suffered from one or more psychological problems (mostly anxiety, 12.6%) following abortion [24].

As for the limitations of this study, a significant setback was the data collection method. Originally, it was planned that all data would be collected in OBGYN clinics in Jeddah. However, due to the consequences of the current pandemic, we were unable to do so and had to use the online distribution of the questionnaire, which may have led to bias.

## Conclusions

In conclusion, this study aimed to evaluate the prevalence of depression, anxiety, and stress among pregnant women in Jeddah, Saudi Arabia, and to assess risk factors. Several risk factors were associated with these mental issues, including mode of last delivery, history of C-section delivery, and spouse/family support during pregnancy. The results of our study concluded that a significant percentage of women suffered from psychological problems, which proves that pregnant women need more support to reduce their levels of psychological symptoms and help them adapt to pregnancy-related changes. This study serves as a basis for the exploration of psychological issues in women during pregnancy, specifically in the Western region of the country, which could aid future research in the exploration of the exact risk factors for mental illness in pregnant women.

It is recommended that mental health support be available to perinatal women in basic antenatal care clinics. This would help improve fetal and maternal outcomes. Health authorities should focus on providing knowledge about mental health problems using appropriate methods for promoting the mental well-being of pregnant women in Jeddah, and they should pay more attention to high-risk groups. Moreover, community mental health care should be made accessible to people who are at high risk.
